# Trial of ligation versus coagulation of lymphatics in dynamic inguinal sentinel lymph node biopsy for staging of squamous cell carcinoma of the penis

**DOI:** 10.1308/003588412X13171221591899

**Published:** 2012-07

**Authors:** S La-Touche, B Ayres, W Lam, H Alnajjar, M Perry, N Watkin

**Affiliations:** St George’s Healthcare NHS Trust,UK

**Keywords:** Sentinel lymph node biopsy, Diathermy, Ligation, Lymphocele

## Abstract

**INTRODUCTION:**

Te principal advantage of dynamic sentinel lymph node biopsy (DSNB) over modified inguinal node dissection is the lower complication rate. The aim of this study was to identify factors associated with short-term complications of DSNB in order to lower morbidity of the procedure.

**METHODS:**

Retrospective and prospective cohort studies were performed on patients undergoing DSNB between April 2005 and March 2010. Patients were categorised into three groups of 50 (from a total of 250 patients on the database). The patients of Group A, on whom ligaclips were the lymphovascular control technique, were compared with those of Group B, in whom diathermy was used. Incision length, operative time, number of nodes removed, antibiotics and co-morbidities were recorded. A prospective study on Group C, using ligaclips, was also performed.

**RESULTS:**

Groups A (88 groins), B (75 groins) and C (68 groins) were explored with complication rates of 5.7%, 24.0% (*p*=0.0018) and 8.8% (*p*=0.0277). Co-morbidities, antibiotics (co-amoxiclav 1.2g intravenous as per protocol) and the mean number of nodes removed were similar in all groups. The mean incision length was 4.1cm (standard deviation [SD]: 1.0cm) for Group A, 5.6cm (SD: 1.0cm) for Group B (*p*=0.0001) and 5.6cm (SD: 0.8cm) for Group C (*p*=0.979). The mean operative times for Groups A, B and C were 15.8 (SD: 8.1), 19.3 (SD: 7.4) (*p*=0.0043) and 22.1 (SD: 7.7) (*p*=0.0301) minutes respectively.

**CONCLUSIONS:**

Lymphovascular control with diathermy is associated with a statistically higher short-term complication rate compared with ligaclip usage (ie ‘permanent’ ligation). Lymphocoeles are the principal complication and can result in delayed wound infection and breakdown. A small but statistical increase in operative time and wound length is likely to be related to registrar training.

Squamous cell carcinoma of the penis is a rare disease with an incidence of less than 1 per 100,000 in Europe and the US.^1,2^ The term ‘sentinel node’ was first described by Gould in 1960 during observations in patients with parotid cancer and further developed as a concept in penile cancer by Cabanas in 1977.^3,4^ Introduced in 1994, dynamic sentinel lymph node biopsy (DSNB) for penile carcinoma involves injection of nanocolloid at the base of the penis followed by scintigraphy, with radioactive tracer seen to drain into the left and right inguinal basins. The sentinel nodes are marked and, with the aid of a subdermal injection of patent blue dye, removed through a small inguinal incision.

There has been much controversy surrounding DSNB, which has previously demonstrated a high false negative rate (20–40%).^5–7^ However, more recent data report a false negative rate of approximately 5%.^8^ Morbidity in traditional inguinal lymph node dissection has been reported by various authors as 30–50%^9,10^ with mortality of up to 3%.^11^ Complications include wound infection, skin necrosis, dehiscence and lymphocoele.^9^ The principal advantage of DSNB over modified inguinal node dissection is the claimed lower complication rate, both in the short and long term, as well as its reliability.^8,12–14^

European Association of Urology guidelines suggest the use of DSNB in patients with intermediate and high risk disease and clinically inguinal node negative disease.^15^ Since 2004, our unit has preferentially performed DSNB to reduce complications in the management of node negative squamous cell carcinoma of the penis. In early 2009 we became concerned about an increase in complications. These were mostly in patients who presented after 2–4 weeks to their local hospitals with a spectrum of problems including lymphocoeles, wound infection and wound breakdown. We suspected that this might have been due to our use of diathermy control of lymphatics, which had gradually become our practice due to convenience compared with the use of ligaclips in our initial patient group

The objective of this study was to identify any factor associated with short-term complications of DSNB in order to potentially lower morbidity of the procedure at a UK tertiary referral centre for penile cancer.

## Methods

The technique of sentinel lymph node biopsy used in this study was described by Cabanas in 1977 and further developed by Horenblas *et al*.^4,13^ In summary, it involves injection of nanocolloid at the base of the penis followed by lymphoscintigraphy, with radioactive tracer seen to drain into the left and right inguinal basins. The sentinel nodes are marked and, with the aid of patent blue dye, injected circumferentially around the penile shaft. The nodes are removed through a small inguinal incision.

Data had been collected during surgery since 2004 on a specifically designed data sheet. They had then been entered on a database that was updated with complications if they occurred post-operatively in hospital or on review at clinic. A partly retrospective and partly prospective cohort study was performed on patients who underwent DSNB between April 2005 and March 2010. There were 250 patients on the database in total.

Patients were categorised into three groups. Group A consisted of the first 50 patients on whom ligaclips were the lymphovascular control technique used. This group was compared with a second cohort of 50 patients (Group B), in whom coagulation diathermy was primarily used. A prospective study was then performed on another 50 patients with ligaclips (Group C). The primary outcome was the rate of short-term complications of lymphocoele, haematoma, wound infection and wound breakdown. However, we contacted the patients in Cohort A to be sure that they had not suffered delayed complications that were unknown to us. The patients in Group B were also contacted to verify complications.

Confounding variables including incision length, operative time, number of nodes removed, antibiotic usage, grade of surgeon and co-morbidities (smoking, diabetes, hypertension) were recorded. The subsequent analysis was based on patients who had complete data sets only. Prism® (GraphPad Software Inc, La Jolla, CA, US) was used to analyse the data with Fisher’s exact test and t-tests.

## Results

In Group A, 88 inguinal basins were explored with a complication rate of 5.7% while in Group B, 75 groins were explored with a 24.0% complication rate (*p*=0.0018), mostly lymphocoeles with/without infection. In Group C, 68 inguinal basins were explored with a complication rate of 8.8% (*p*=0.0277, vs Group B) (Table 1).

**Table 1:** Summary of results

Group	Inguinal basins	Complication rate	Lymphocoele	Haematoma	Wound infection	Wound breakdown	Mean incision length	Mean operative time	Mean nodes per groin
A	88	5.7%	3.4%	1.1%	1.1%	–	4.1cm	15.8 mins	1.9
B	75	24.0%	14.7%	–	8.0%	1.3%	5.6cm	19.3 mins	2.1
C	68	8.8%	5.9%	–	2.9%	–	5.6cm	22.1 mins	2.0

The mean incision length was 4.1cm (standard deviation [SD]: 1.0cm) for Group A, 5.6cm (SD: 1.0cm) for Group B and 5.6cm (SD: 0.8cm) for Group C (*p*=0.0001 A vs B and *p*=0.979 B vs C). The mean operative time was 15.8 minutes (SD: 8.1 minutes) for Group A, 19.3 minutes (SD: 7.4 minutes) for Group B and 22.0 minutes (SD: 7.7 minutes) for Group C (*p*=0.0043 A vs B and B vs C). The mean number of nodes removed was 1.9 in Group A, 2.1 in Group B and 2.0 in Group C (Table 1).

Co-morbidities were similar in all groups. Co-amoxiclav (1.2g intravenous) was used as per protocol for prophylaxis in all groups or, if this could not be tolerated, a cefalosporin was used.

## Discussion

The data support our theory that coagulation of lymphatics is only temporary and can result in delayed complications. Lymphatic channels remain closed with clips whereas they may re-open after the use of diathermy when burnt tissue heals. This is supported by other literature, which suggests that as lymph does not contain clotting factors, divided lymphatics never heal spontaneously and therefore cannot be sealed with diathermy. This evidence relates to iliac nodes in renal transplantation and the use of clips or non-absorbable sutures is advocated.^16^ There is also some evidence to suggest that the excessive use of diathermy may increase the risk of lymphocoele formation after pelvic lymph node dissection in prostate cancer.^17,18^

A systematic review published in 2011 analysed the complications of DSNB in the treatment of penile cancer.^19^ Six studies were reviewed, demonstrating a complication rate of 27.3% in inguinal lymph node dissection and 3.6% in sentinel lymph node biopsy (*p*<0.0001). The complications included lymphocoele, seroma, lymphoedema and wound infection. The DSNB complications are similar to those found in our study but the complication rate is slightly lower and there is no consistency or detail regarding the technique of lymph node dissection in these studies.

There are some limitations to our study, including a smaller sample size in Group C, the time of onset of the complications not being measured prospectively and the exclusion of patients with missing data sets. In addition, there is an inability to be certain of the exact nature of the complications in Group B. It was difficult to confirm that a lymph leak preceded a wound infection and subsequent wound breakdown in several cases.

The increase in incision length and theatre time in Groups B and C could be attributed to registrar training whereas in the first cohort procedures were performed principally by the consultant as a new technique was being introduced. It is also possible that for Group C surgeons were taking more care, resulting in longer operative time and fewer complications. However, given that the mean operative time in Group B was greater than that in Group A, we believe that this is unlikely and that this further supports our theory of registrar training time.

Some comparisons can be made with other dynamic sentinel lymph node trials. In the multicentre Groningen international study on sentinel nodes in vulvar cancer, 623 groins in 403 women with T1/T2 vulval cancer were explored.^20^ A total of 264 of these patients had sentinel lymph node biopsy only with a short-term complication rate of 16.2% (principally wound breakdown and cellulitis), which is higher than that reported for our final cohort. Another, single centre study of patients with vulval cancer reported a 5.5% short-term complication rate of mainly lymphocoeles but included only 35 patients.^21^ Neither study provided detail of the method of lymphovascular closure technique.

## Conclusions

From our study it can be concluded that lymphovascular control with diathermy is associated with a higher short-term complication rate in DSNB compared with ligaclips. Lymphocoeles are the principal complication and can result in delayed wound infection and breakdown. A small but statistical increase in operative time and wound length is likely to be related to registrar training.
